# Anthracene-Modified Nanoporous Silica Nanoparticles for ATP Detection and Salivary Diagnostics in Parkinson’s Disease

**DOI:** 10.1021/acsanm.5c05223

**Published:** 2026-03-20

**Authors:** Elcin Ezgi Ahi, Estela Climent, Cansu Beyret, Mehmet Gokhan Caglayan, Burak Coban, Rezzak Yilmaz, Vicente Martí-Centelles, Ramón Martínez-Máñez, Mahmut Durmuş

**Affiliations:** A Department of Chemistry, 52962 Gebze Technical University, Gebze, Kocaeli 41400, Türkiye; B Instituto Interuniversitario de Investigación de Reconocimiento Molecular y Desarrollo Tecnológico (IDM), Universitat Politècnica de València, Universitat de València, Camino de Vera s/n, Valencia 46022, Spain; C CIBER de Bioingeniería Biomateriales y Nanomedicina (CIBER-BBN), Instituto de Salud Carlos III, Valencia 46022, Spain; D Unidad Mixta de Investigación en Nanomedicina y Sensores, Universitat Politècnica de València, Instituto de Investigación Sanitaria La Fe (IIS La Fe), Avenida Fernando Abril Martorell, 106, Valencia 46026, Spain; E Department of Analytical Chemistry, Faculty of Pharmacy, Ankara University, Yenimahalle, Ankara 06560, Türkiye; F Department of Neurology, School of Medicine, Ankara University, Ankara 06100, Türkiye; G Brain Research Center, Ankara University, Altındaǧ, Ankara 06230, Türkiye; H Unidad Mixta UPV-CIPF de Investigación en Mecanismos de Enfermedades y Nanomedicina, Valencia, Universitat Politècnica de València, Centro de Investigación Príncipe Felipe, Avenida Eduardo Primo Yúfera, 3, Valencia 46012, Spain; I Departamento de Química, 16774 Universitat Politècnica de València, Camino de Vera s/n, Valencia 46022, Spain

**Keywords:** anthracenes, atp, MCM-41, optic array sensors, Parkinson’s disease, LDA analysis

## Abstract

ATP is an essential biological molecule, and abnormal levels are linked to various diseases. In this study, we developed an optical sensor array consisting of an anthracene-modified MCM-41 mesoporous nanoparticle-based hybrid organic–inorganic material to detect ATP in a TRIS-HCl buffer (pH 7). To create the sensor, we synthesized MCM-41 silica nanoparticles and functionalized them with aminoanthracene and imidazolium-anthracene groups. The fluorescence responses of the optical sensor array were recorded and analyzed by using a pattern recognition protocol, specifically linear discriminant analysis, to distinguish between ATP, ADP, AMP, and potential interferents. Quantitative regression analysis of ATP in TRIS-HCl was performed using artificial neural networks, which yielded an acceptable error rate of 7.8%. The sensor array was also evaluated for discrimination of Parkinson’s disease (PD) from healthy controls, based on pattern recognition of ATP-related analytes in saliva samples from 24 patients with PD and 23 healthy controls. The results showed that the MCM-41 mesoporous silica nanoparticle-based sensor array can discriminate PD by pattern recognition with 73.7% sensitivity and 83.3% specificity.

## Graphical abstract

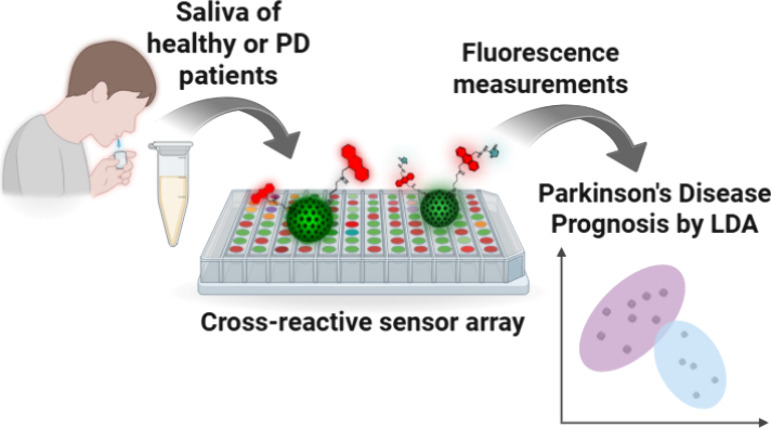

## Introduction

Parkinson’s disease (PD) is the second most common neurodegenerative disorder worldwide, and its global burden is predicted to double from six million cases in 2015 to over 12 million by 2040.^1^ PD is characterized by progressive degeneration of dopaminergic neurons in the substantia nigra, leading to motor symptoms such as tremor, stiffness, bradykinesia (slowness of movement), and impaired balance. By the time symptoms become clinically apparent, approximately 60–80% of dopamine-producing neurons are lost, and surviving neurons frequently contain Lewy bodies—intracellular deposits composed primarily of misfolded protein alpha-synuclein.^2^ According to the recommendations of the International Parkinson and Movement Disorder Society, PD diagnosis relies on clinical evaluation, laboratory tests to exclude other disorders, and brain imaging techniques such as single-photon emission computed tomography (SPECT) and structural magnetic resonance imaging (MRI); however, no definitive diagnostic test is available.^2^

Recently, the α-synuclein seeding amplification assay (SAA) has emerged as a promising approach for PD diagnosis through detection of pathological α-synuclein aggregates in the cerebrospinal fluid (CSF).^3^ Despite its potential, SAA remains limited by a lack of standardization, restricted availability, high cost, and the need for invasive lumbar puncture. These limitations motivate the exploration of alternative, noninvasive analytical approaches aimed at probing disease-related biochemical alterations, rather than establishing standalone diagnostic tools, using accessible biofluids such as saliva.

PD is closely associated with impaired brain energy metabolism, and the age-related decline in glucose utilization in the brain is considered a major risk factor for disease development.^4^ Deficiencies in nicotinamide adenine dinucleotide (NAD) and adenosine 5′-triphosphate (ATP) have been reported in PD brains, and mitochondrial dysfunction is recognized as an early pathogenic event.^5,6^ Environmental toxins induce mitochondrial dysfunction, while several PD-associated genetic mutations and aging-related mitochondrial impairment contribute to reduced ATP production.^4^ Although PD was once thought to be confined to the central nervous system, it is now well-established that PD pathology also involves peripheral tissues, including the gastrointestinal tract, skin, and salivary glands.^7–9^ Among these, saliva offers an easily accessible, cost-effective, and noninvasive biosample that has been previously shown to reflect PD-related molecular changes.^10,11^ In addition, ATP-sensitive receptors play a role in salivary gland secretion, and the salivary enzyme ATP13A2 (ATPase cation transporting 13A2) has emerged as a potential PD-associated biomarker suggesting that ATP-related biochemical changes may be reflected in saliva.^7,12^

ATP is an essential biological molecule involved in cellular energy transfer, intracellular signaling, neurotransmission, and nucleic acid synthesis.^13–21^ Dysregulated ATP levels have been linked not only to PD but also to cancer, viral infections, and hypoxia.^4,5,20,22–24^ Numerous analytical techniques have been developed for ATP detection in environmental and biological samples, including bioluminescence, chemiluminescence, colorimetry, potentiometry, fluorometry, high-performance liquid chromatography (HPLC), and nuclear magnetic resonance (NMR).^25–36^ While reliable, these methods often require expensive reagents, controlled assay conditions, sophisticated instrumentation, or extensive sample preparation, limiting their suitability for rapid and decentralized analysis.

Optical chemosensors based on small molecular probes offer an attractive alternative due to their simplicity, real-time response, and uncomplicated instrumentation requirements.^37^ Among fluorescent probes, anthracene derivatives are widely used because of their high fluorescence quantum yields, photostability, extended π–π conjugation, and ease of chemical modification.^38^ However, free molecular probes often exhibit limited robustness and selectivity in complex biological matrices.

Integration of molecular probes into nanostructured materials provides an effective strategy to overcome these limitations. In particular, mesoporous silica nanoparticles (MSNs) are distinguished by their high surface-to-volume ratios, ordered and tunable pore architectures, large internal surface areas, and versatile surface chemistry.^39,40^ These nanoscale features enable high-density probe immobilization, multivalent and cooperative analyte interactions, signal amplification, and improved stability, which are especially advantageous in complex media such as saliva.^41–43^ Among MSNs, MCM-41-type materials are notable for their uniform cylindrical mesopores, narrow pore size distribution, high specific surface area, and well-defined ordered framework.^44^

Previous studies have shown that anthracene-functionalized MCM-41 materials exhibit enhanced ATP responses compared to free probes, underscoring the importance of nanoscale organization and surface charge density.^45^ Nevertheless, ATP recognition in such systems is often dominated by nonspecific electrostatic interactions, resulting in limited selectivity.^46^

Rather than relying on highly specific molecular receptors, cross-reactive optical sensor arrays provide an alternative strategy by exploiting differential interactions between analytes and multiple sensing elements to generate characteristic response patterns or “fingerprints”.^47–49^ When coupled with multivariate statistical methods such as principal component analysis (PCA), hierarchical cluster analysis (HCA), and linear discriminant analysis (LDA), these fingerprint-based approaches enable pattern recognition and sample discrimination, even in complex biological matrices.^46,47,50–52^

Based on this concept, we report the design of two MCM-41-based mesoporous silica nanoparticle hybrid materials functionalized with imidazolium-anthracene (**S2**) and aminoanthracene (**S3**) moieties. These materials were integrated into an optical cross-reactive sensor array that generates ATP-related response fingerprints. Using LDA, the array discriminates ATP, ADP, AMP, H_2_PO_4_^–^, acetate (AcO^–^), benzoate, chloride (Cl^–^), and fluoride (F^–^) in both the TRIS-HCl buffer solution and saliva. Furthermore, the sensor array enables pattern recognition-based discrimination of saliva samples from PD patients and healthy controls, illustrating the potential of mesoporous silica nanoparticle-based hybrid sensor arrays as a tool for probing PD-associated biochemical patterns in saliva.

## Experimental Section

### Materials

Tetraethyl orthosilicate (TEOS), cetyltrimethylammonium bromide (CTAB), and sodium hydroxide (NaOH) were purchased from Sigma-Aldrich. Protease inhibitor cocktail tablets (EDTA-free) were purchased from Roche. Sodium orthovanadate (CAS number 13721-39-6) was purchased from AppliChem. Anthracene-9,10-dicarboxyaldehyde, 9-anthraldehyde, and 2-hydrazino-2-imidazoline hydrobromide were purchased from Sigma-Aldrich. Sodium dihydrogen phosphate (NaH_2_PO_4_), sodium pyrophosphate, (Na_4_P_2_P_7_), sodium fluoride (NaF), and adenosine 5′-monophosphate (AMP) and cytidine triphosphate (CTP) were purchased from Merck. Adenosine 5′-diphosphate (ADP) and adenosine 5′-triphosphate (ATP) were purchased from Thermo Scientific. Sodium acetate-3-hydrate and sodium benzoate were purchased from Riedel-de Haen.

### Equipment

1H NMR spectra was recorded on a Bruker FT-NMR Avance 400 (Ettlingen, Germany). TEM images were acquired using a JEOL TEM-1010 electron microscope working at 100 kV. SEM analysis was performed using a Quanta 400F field emission SEM. Powder X-ray diffraction (PXRD) measurements were performed using a Seifert 3000TT diffractometer using Cu Kα radiation. N_2_ adsorption–desorption isotherms were recorded using a Micromeritics TriStar II Plus automated analyzer. Z-potential measurement studies were performed using a ZetaSizer Nano ZS (Malvern). Fluorescence measurements were performed with a BMG Clariostar microplate reader.

### Synthesis of the Imidazolium Derivative and the Silica Nanoparticles

#### Synthesis of the Imidazolium Derivative (**1**)

Imidazolium derivative molecule **1** was synthesized following the procedure reported in the literature.^53^ Briefly, 25 mg (0.11 mmol) of anthracene-9,10-dicarboxaldehyde and 20 mg (0.11 mmol) of 2-hydrazino-2-imidazoline hydrobromide were dissolved in 20 mL of anhydrous ethanol. After heating the mixture, 8 μL of concentrated HCl was added, and the mixture was stirred under reflux for 2 h. The resulting reaction mixture was then concentrated to obtain the probe **1** as an orange precipitate (Scheme 1). The ^1^H NMR spectrum of **1** is given in the Supporting Information in Figure S1.

**Scheme 1:**
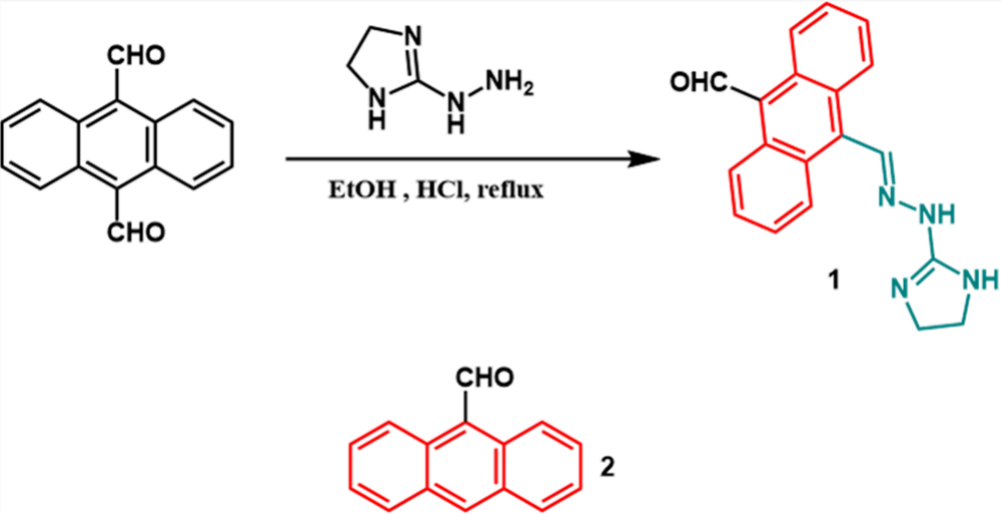
Synthesis of the Imidazolium Derivative (**1**) and the Molecular Structure of 9-Anthracenaldehyde (**2**)

#### Synthesis of Silica Nanoparticles (MCM-41) (**S0**)

The MCM-41 mesoporous silica nanoparticles were synthesized by following the procedure reported in the literature.^54^ Cetyltrimethylammonium bromide (CTAB, 1.00 g, 2.74 mmol) was dissolved in 480 mL of deionized water. After complete dissolution of CTAB, 3.5 mL of a 2 M NaOH solution was added. Once the temperature reached 80 °C, tetraethyl orthosilicate (TEOS, 5 mL) was added dropwise to the mixture. The mixture was allowed to stir for 2 h at 80 °C to give a white precipitate.

After 2 h, the mixture was allowed to cool to room temperature. The resulting solid was filtered, washed with deionized water, and dried at 65 °C (MCM-41, as-synthesized). To obtain the final porous material (MCM-41, **S0**), the as-synthesized solid was calcined at 550 °C overnight to remove the template phase.

#### Grafting of **S0** with (3-Aminopropyl)­triethoxysilane (**S1**)

The solid **S1** was synthesized using a well-known grafting method.^55^ Briefly, 100 mg of **S0** was suspended in 8 mL of anhydrous toluene, and (3-aminopropyl)­triethoxysilane (APTES) (1.2 mmol) was added. The suspension was stirred overnight at 90 °C and then filtered. The resulting white solid was washed with toluene and ethanol and dried at 38 °C to give **S1**.

#### Functionalization of **S1** with Imidazolium Hydrazone-Based Anthracene (**S2**)

80 mg of **S1** was immersed in 7 mL of ethanol and 105 mg (0.332 mmol) of **1** (imidazolium-anthracene derivative) was added. The mixture was stirred at 45 °C under an argon atmosphere overnight. The resulting solid was filtered and washed with ethanol to remove any excess of compound **1**. The obtained product was suspended in 6 mL of absolute ethanol, and NaBH_4_ (0.34 mmol) was added. The mixture was stirred at room temperature for 24 h. Then, the solid **S2** was washed with ethanol and 0.1 M HCl until the pH reached approximately 5–6. Finally, the solid was washed again with ethanol and dried to obtain final product of **S2** (Figure 1).^55^

**Figure 1:**
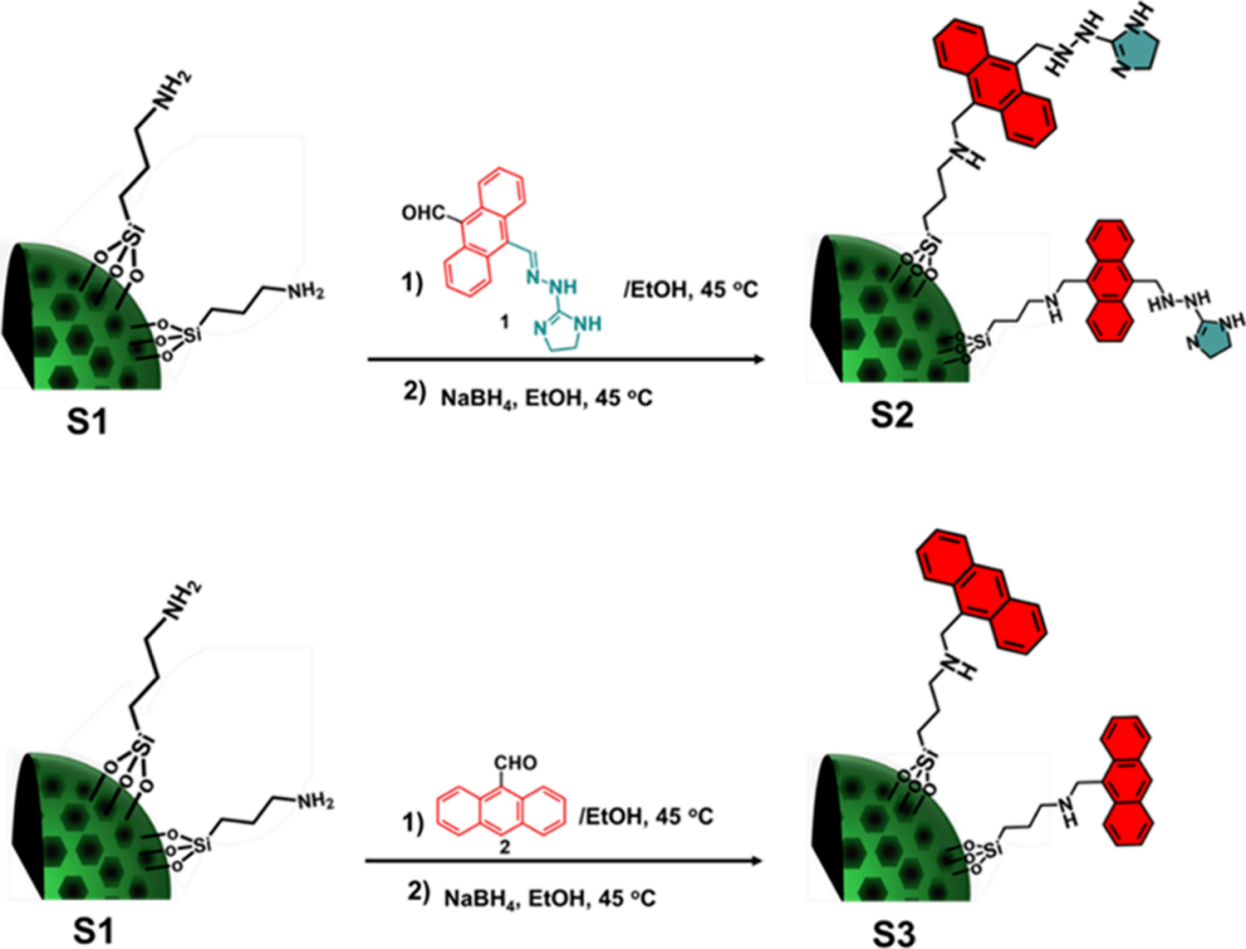
Synthesis of **S2** (upper) and **S3** (bottom): first, calcined MCM-41 was grafted with APTES to yield **S1**, and then, **S1** was reacted with imidazole-anthracene and anthracene derivatives to yield **S2** and **S3**, respectively.

#### Functionalization of **S1** with 9-Anthracenaldehyde (**S3**)

For the synthesis of **S3**, 80 mg of **S1** was suspended in ethanol, and 62 mg (0.3 mmol) of 9-anthracenaldehyde (**2**) was added. The mixture was stirred at 45 °C under an argon atmosphere overnight following the same procedure as described for the synthesis of **S2**. After the final washing step with ethanol, solid **S3** was obtained (Figure 1).

### Qualitative and Quantitative Analysis Based on Cross-Reactive Sensor Arrays of **S2** and **S3**

#### Qualitative Analysis in Buffer Solution

Qualitative analysis was performed based on a cross-reactive sensor array consisting of sensor elements of **S2** and **S3**. Stock solutions of ATP, ADP, AMP, H_2_PO_4_^–^, acetate, benzoate, fluoride, and chloride were prepared at a concentration of 10 mM in TRIS-HCl buffer (pH 7, 20 mM). Solid sensors **S2** and **S3** were prepared at a concentration of 1 mg/mL as stock solutions in TRIS-HCl buffer (pH 7, 20 mM).

Fluorescence measurements were performed in 96-well microplates using a BMG Clariostar microplate reader. In a black 96-well microplate, 10 μL of a **S2** or **S3** stock solution (1 mg/mL) was dispensed into individual wells. Subsequently, 20 μL of each analyte solution was added to the corresponding wells. For blank measurements, 10 μL of the **S2** or **S3** stock solution was added to wells without the analyte. The final volume in each well was adjusted to 200 μL with TRIS-HCl buffer solution (pH 7). The construction of the microplate for the sensor array can be seen in Figure S2.

After fluorescence measurements, the emission values of 9 replicates for each analyte were used for the LDA. All data sets were used as the training set, while leave-one-out cross-validation (jackknifing) was used in the validation of LDA.

#### Qualitative Analysis in Saliva

Qualitative analysis of saliva samples was performed by following the same procedure as in the buffer solution. Prior to constructing the sensor arrays on two separate microplates (for **S2** and **S3**), the saliva samples were prepared. In eight separate Eppendorf tubes, 500 μL of pooled healthy saliva was transferred, followed by the addition of 15 μL of sodium orthovanadate (2 mM stock solution) and 30 μL of the protease inhibitor. Subsequently, 223 μL of each analyte (10 mM stock concentration) was added to the saliva samples, and the final volume in each tube was adjusted to 2 mL using TRIS-HCl buffer (pH 7). For blank measurements, 500 μL of saliva was diluted to 2 mL with a buffer after the addition of the inhibitors.

Sensor array construction involved adding 10 μL of solid **S2** to 90 μL of each prepared saliva sample (including those with different analytes) in a 96-well microplate. A separate microplate was prepared in the same manner using solid **S3**. Each saliva sample, including those with different analytes, was analyzed in nine replicates per plate. Following the fluorescence measurements, the emission values from the nine replicates for each analyte were used for LDA. The entire data set was used as the training set, while leave-one-out cross-validation (jackknifing) was used for validation of the LDA model.

#### Quantitative Analysis in Buffer Solution

To perform the quantitative analysis, different volumes (between 5 and 100 μL) of a stock solution of ATP (10 mM) were transferred into the microplate, which includes 10 μL of modified MCM-41 solids into each well, and then subsequently diluted to 200 μL into each well with TRIS-HCl buffer solution (pH 7, 20 mM) to obtain concentrations ranging from 0.25 to 5 mM. For each concentration, 3 replicates were prepared in a microplate for both sensor solids **S2** and **S3**.

Following the fluorescence measurement, the fluorescence emission data were used for regression analysis by artificial neural networks (ANN), enabling accurate prediction of ATP concentrations within the tested range. The concentrations of ATP ranging from 0 to 5 mM were used as a training data set. After that, the validation was performed by using the concentrations of 0.74 and 1.5 mM of ATP.

#### Quantitative Analysis in Saliva Samples

The standard addition method was employed across ATP concentrations ranging from 0.25 to 5 mM. In a 2 mL Eppendorf tube, 500 μL of saliva was transferred, followed by the addition of 15 μL of sodium orthovanadate (2 mM stock solution) and 30 μL of protease inhibitor. To achieve the desired ATP concentration, appropriate volumes of ATP stock solution were added and the final volume was adjusted to 2 mL with TRIS-HCl buffer solution (pH 7). For each concentration, nine replicates were prepared in microplates for both sensor solids **S2** and **S3**.

Following the fluorescence measurement, the fluorescence emission data were subjected to regression analysis using ANN, enabling accurate prediction of ATP concentrations within the tested range. The concentrations of ATP ranging from 0 to 5 mM were used as a training data set. After that, the validation was performed by using the concentrations of 0.74 and 1.5 mM of ATP.

### Participants and Clinical Assessments

Participant recruitment and clinical examinations were conducted by movement disorder specialists at the Movement Disorder Unit, Department of Neurology, Ankara University School of Medicine. Between January 2022 and August 2023, a total of 24 patients with PD and 23 age-matched healthy controls (all aged >60 years) were enrolled in the study. PD diagnosis was established based on the UK Brain Bank criteria.

Comprehensive data, including demographic information, risk factors, and clinical scores, were collected for all patients. Motor evaluations and disease severity were evaluated by the Movement Disorders Society-Unified Parkinson’s Disease Rating Scale (MDS-UPDRS) and Hoehn and Yahr scale, respectively. Additionally, nonmotor symptoms—including autonomous dysfunction, mood, cognition, and quality of life—were evaluated in all patients. Healthy controls were selected from individuals without neurodegenerative disease based on clinical assessment.

A standardized protocol was followed for saliva sample collection from all participants. Prior to sample collection, detailed information regarding each participant’s oral health status was obtained. Only individuals who had fasted overnight for at least 8 h were eligible for inclusion. Participants who had brushed their teeth, chewed gum, or used oral sprays within 2 h prior to collection were excluded. Additional exclusion criteria included smoking within 10 min of sample collection and the use of lipstick or similar cosmetic products on the day of sampling.

Saliva samples were collected using the passive drool method. Each participant was instructed to drool into a sterile cup three times, and approximately 3–6 mL of saliva was collected per participant. Following sample collection, samples were immediately stored at −80 °C (Thermo Scientific) until further analysis.

All participants provided written informed consent prior to participation. The study was approved by the Ethics Committee of Ankara University School of Medicine (ethics committee approval i9-583-21), and all procedures were conducted in accordance with the Declaration of Helsinki. The clinical characteristics of patients with PD are given in Table S1.

### Array Sensing of Saliva for Pattern Recognition-Based Discrimination of Parkinson’s Disease

For array sensing of PD, saliva samples were diluted in a TRIS-HCl buffer solution (20 mM, pH 7.0). Specifically, 250 μL of each saliva sample was diluted to a final volume of approximately 980 μL. The diluted samples were then treated with 7.5 μL of sodium orthovanadate (2 mM stock solution) as a phosphatase inhibitor along with 15 μL of a 7× protease inhibitor cocktail.

Fluorescence measurements were performed by using a black 96-well microplate. Into each well, 10 μL of the MCM-41-based sensor probes **S2** and **S3** (1 mg/mL stock solution) was added. Subsequently, 90 μL of the inhibitor-treated saliva samples was transferred into the wells. Fluorescence measurements were carried out by using a microplate reader with an excitation wavelength of 369 nm for **S2** and 380 nm for **S3**. Each saliva sample was analyzed in quadruplicate with both **S2** and **S3** sensors.

### Fluorescence Measurements

Fluorescence measurements were performed using a microplate reader operated in well-scan mode (spiral scan, 6 mm diameter), which averages the fluorescence signal over multiple positions within each well to minimize local inhomogeneities arising from dispersed solid suspensions. Prior to measurement, all samples were vortexed to ensure uniform dispersion, and the microplates were shaken for 40 s at 100 rpm. The number of flashes per well was set to 106, with a gain value of 1500 and a focal height of 7.4 mm. Independent measurements were obtained using nine replicate wells per condition, which were treated as independent sensing units in the sensor array.

Fluorescence excitation was performed at 369 nm for **S2** and 380 nm for **S3**. Emission intensities were collected at selected wavelengths corresponding to emission maxima (420 and 520 nm) and shoulder regions (401, 444, and 474 nm). These wavelengths were chosen to capture both monomer- and excimer-type emission features of the anthracene fluorophores.

Variations in host–guest interactions and surface aggregation induced by different analytes modulate the relative contributions of these emission bands, thereby generating distinct spectral response patterns suitable for multivariate discrimination.

## Results and Discussion

### Characterization of Prepared Nanoparticles

Characterization of the MCM-41 mesoporous silica nanoparticles, including as-made and calcined material (**S0**), as well as the functionalized derivatives (**S1**, **S2**, and **S3**, Figure 1), was performed using standard procedures such as powder X-ray diffraction (PXRD), nitrogen adsorption–desorption (N_2_ adsorption–desorption) isotherms, and transmission electron microscopy (TEM). PXRD patterns of the as-made MCM-41 and the calcined MCM-41 solids (**S0**) (Figure 2A) revealed a characteristic intense peak at ca. 2θ = 2°, which corresponds to the (100) reflection, consistent with the presence of a hexagonal MCM-41-type mesostructure. Additionally, both materials showed four low-angle reflections, which can be indexed to the (100), (110), (200), and (210) planes, typical of a well-ordered hexagonal array. The calculated unit cell parameter (*a*_0_) was 38.56 Å, with a corresponding *d*_100_ spacing of 33.39 Å for the calcinated solid **S0**. The XRD pattern of the calcined MCM-41 (**S0**) solid exhibited a slight shift of the (100) peak (Figure 2A) attributed to the condensation of the silanol groups during calcination. This structural change resulted in an approximate cell contraction of 2.45 Å.

**Figure 2:**
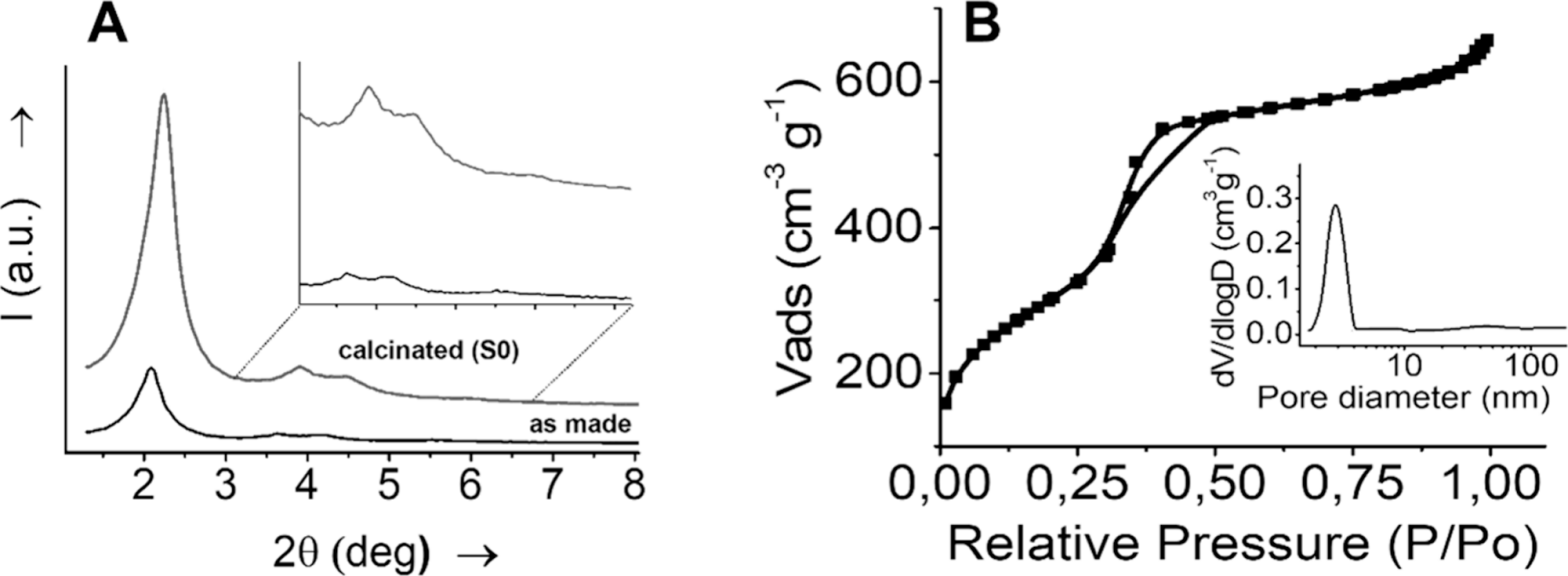
(A) PXRD patterns of the as-made MCM-41 and calcined MCM-41 solids (**S0**) and (B) N_2_ adsorption–desorption isotherms of **S0**.

The N_2_ adsorption–desorption isotherm of **S0** (Figure 2B) exhibits features typical of mesoporous materials. At low relative pressures (from 0 to ca. 0.3 *P*/*P*_0_), a quasilinear increase in the adsorbed volume is observed, indicating monolayer–multilayer adsorption. The slope in this region is related to the specific surface area, which is 1101.53 m^2^ g^–1^ for **S0** calculated from the Brunauer–Emmett–Teller (BET) model. A sharp increase in the adsorbed volume around 0.30–0.40 *P*/*P*_0_ was observed. This curve corresponds to a type IV isotherm, in which the observed step indicates nitrogen condensation inside the mesopores. From the PXRD, porosimetry, and TEM studies, the *a*_0_ cell parameter (3.85 nm), pore diameter (3.1 nm), and value for the wall thickness (0.75 nm) were calculated for **S0**. The total pore volume, calculated from the amount of nitrogen adsorbed at the plateau region of the isotherm (assuming complete pore filling), was found to be 0.93 cm^3^ g^–1^ (Table 1). The calculation was performed by using the Barret, Joyner, and Halenda (BJH) model on the adsorption branch of the isotherm. In the solids, the existence of uniform cylindrical mesopores is suggested by the absence of a hysteresis loop in this interval and the narrow BJH pore distribution.

**Table 1:** BET Specific Surface Values, Pore Volumes, and Pore Sizes Calculated from N_2_ Adsorption–Desorption Isotherms for Selected Materials

sample	SBET [m^2^ g^–1^]	pore volume [cm^3^ g^–1^]	pore size [nm]
**S0**	1101.53	0.93	3.1

TEM images of **S0** (Figure 3D) confirm the presence of the typical channel structure of the MCM-41. The particles were observed to be spherical in shape with diameters of 76 ± 18 nm. Scanning electron microscopy (SEM) images of the calcined MCM-41 (**S0**), as well as the functionalized solids **S2** and **S3**, reveal a spherical morphology for all samples (Figure 3A–C). Thermogravimetric analysis (TGA) of MCM-41 solids **S0**–**S3** is given in Figure S3, which shows a weight loss between 25 and 165 °C because of elimination of water and solvent molecules, between 165 and 700 °C related with the decomposition of organic matter, and between 700 and 900 °C due to the condensation of silanol groups, and it is well-matched with the typical TGA curve. The loss of mass was calculated from the curves for **S0** (calcined), **S1**, **S2**, and **S3** (Table S2). Contents of APTES, imidazolium-anthracene, and anthracene moieties were determined having in mind the organic mass lost on the materials, obtaining functionalization of 2.35 mmol g solid^–1^ APTES moieties for **S1**, 0.748 mmol g solid^–1^ of anthracene with imidazolium hydrazone for **S2**, and 1.72 mmol g solid^–1^ of anthracene groups for **S3**.

**Figure 3:**
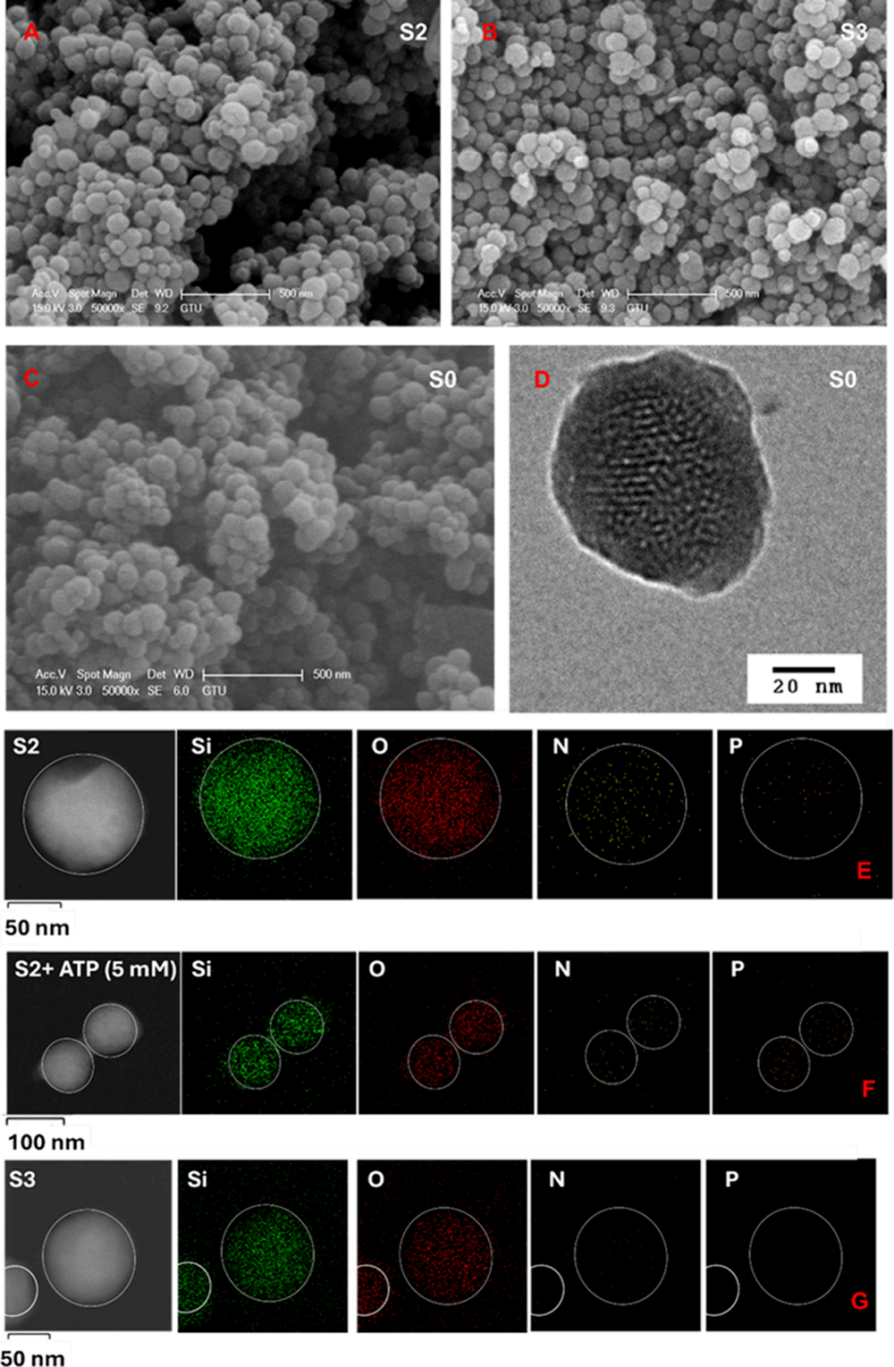
SEM images of (A) **S2**, (B) **S3**, and (C) **S0**; TEM image of (D) **S0**; and STEM-EDX maps of atomic composition (wt %) for (E) **S2**, (F) **S2** + ATP (5 mM), and (G) **S3**.

Additionally, scanning transmission electron microscopy coupled with energy-dispersive X-ray spectroscopy (STEM-EDX) allowed the determination of elemental compositions of **S0**, **S2**, and **S3** (see Table S3 and Figures S4–S6). As expected, calcined MCM-41 (**S0**) consists of SiO_2_. Following surface modification, an increase in the atomic percentages of nitrogen (N) and carbon (C) was observed, confirming the successful functionalization of the mesoporous silica with organic moieties. Moreover, STEM-EDX maps of atomic composition (wt %) were also obtained for **S2** (Figure 3E), **S3** (Figure 3G), and **S2** in the presence of 5 mM ATP, observing an increase in the presence of P due to the interaction of ATP with **S2** (Figure 3F).

Dynamic light scattering (DLS) and ζ-potential analysis allowed us to follow size and surface charge changes during the synthesis of solids **S2** and **S3**. In the presence of ATP, the hydrodynamic diameter increased in both solids, **S2** and **S3**, from 241 ± 26 to 373 ± 20 nm and from 202 ± 12 to 287 ± 40 nm, respectively. Regarding the ζ-potential, **S0** showed typical negative values of MSNs due to the presence of silanol groups (−25.5 mV), which increased in **S2** and **S3** (36 and 38 mV for **S2** and **S3**, respectively). However, the ζ-potential value decreased to slightly negative values (−6 and −8 mV for **S2** and **S3**, respectively) after interaction with ATP, which is negatively charged at a neutral pH.

### Experimental Setup and Fluorescence Measurement

The two materials, i.e., imidazolium-anthracene-functionalized (**S2**) and amino-anthracene-functionalized (**S3**), were used for the discrimination and quantification of anions, including ATP. Preliminary sensing studies were conducted using a microplate reader for fluorimetric titration of both solids **S2** and **S3** with different concentrations of ATP in a 96-well microplate. Aliquots of 10 μL of each material (**S2** and **S3**) were mixed with ATP solutions in TRIS:HCl buffer (pH 7, 20 mM), with concentrations ranging from 0.3 to 5 × 10^3^ μM.

The fluorescence spectra of solids **S2** (Figure 4A) and **S3** (Figure 4B) exhibited a decrease in intensity at 420 nm and a concurrent increase in the intensity of a new emission band around 520 nm upon ATP addition.

**Figure 4:**
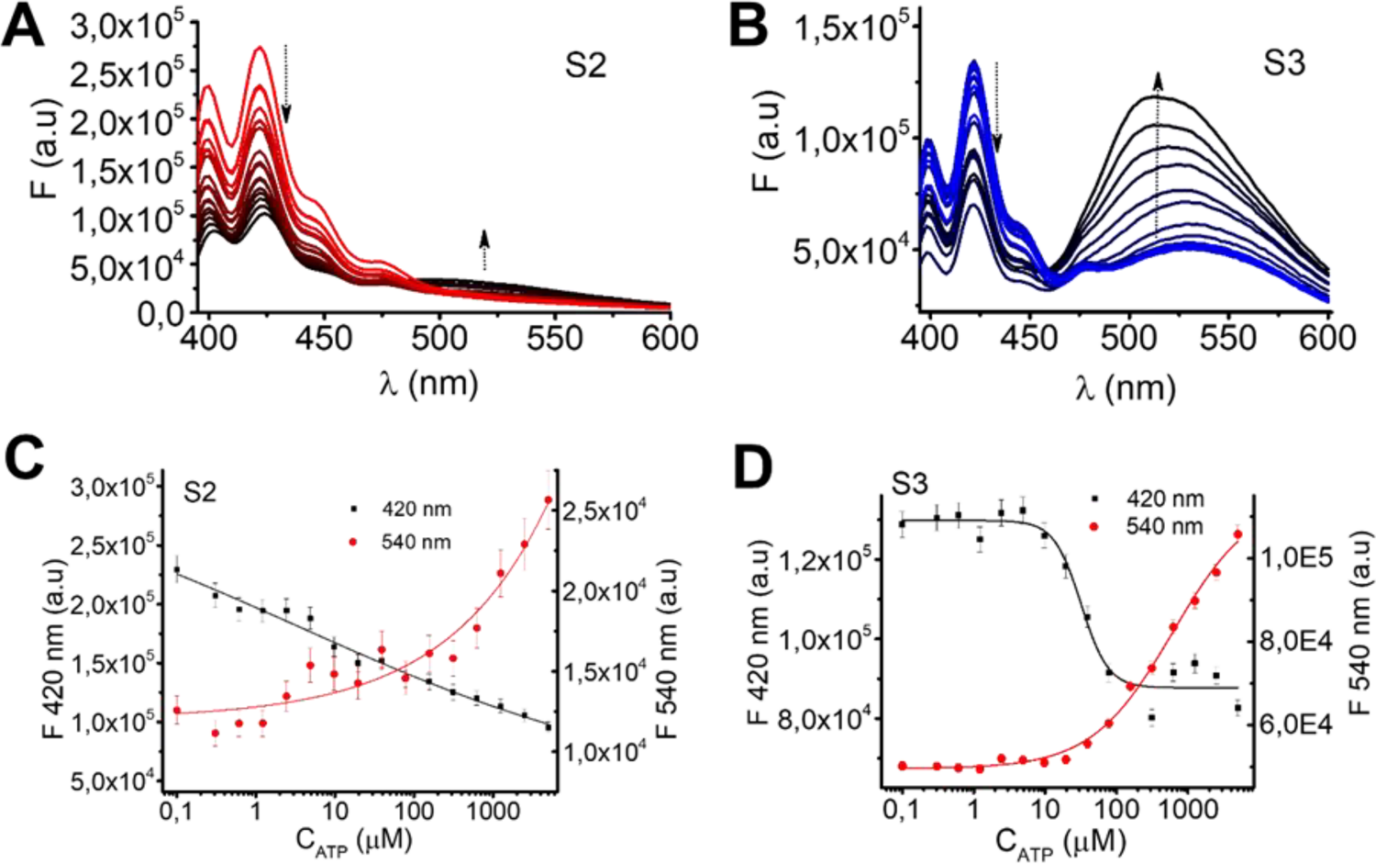
(A) Fluorescence emission titration curves of **S2** (7.48 × 10^–4^ mmol mg^–1^) with ATP, (B) fluorescence emission titration curves of **S3** (1.72 × 10^–3^ mmol mg^–1^) with ATP in TRIS-HCl (pH 7.20 mM), (C) calibration curves for **S2** at 420 and 540 nm, and (D) calibration curves for **S3** at 420 and 520 nm.

Titration studies allowed calculating detection limits of 3 ± 1 and 22 ± 3 μM for ATP for sensors **S2** and **S3**, respectively (Figure 4C,D). **S2** showed better sensitivity than **S3**, which can be explained by the presence of additional imidazole-hydrazone functional groups on the central anthracene fluorophore. Furthermore, **S2** also demonstrated better sensitivity toward ATP when compared with the free bisantrene probe reported by Farshbaf and Anzenbacher, which showed ATP detection within a dynamic range of 1–12 mM.^53^

In related work, Descalzo et al. developed hybrid materials based on MCM-41 by grafting aminoanthracene groups onto mesoporous silica for the detection of ATP.^45^ A comparative analysis revealed that, while the free probe exhibited a low complex stability constant, the aminoanthracene-grafted MCM-41 nanoparticles showed an enhanced response to ATP. This improvement was attributed to the presence of approximately 3 × 10^16^ ammonium groups, enabling the material to act as a massive polyammonium supermolecule in water at pH 2.8.

In the same study, Descalzo et al. also demonstrated the effect of porosity on the sensing performance. An analogous sensing experiment was conducted using nonporous commercial silica fume matrices modified with aminoanthracene. This modified nonporous silica was able to detect ATP at a concentration 100 times lower than that detectable by MCM-41 mesoporous solids. The characteristic periodicity of MCM-41-type mesoporosity, which can be conveniently modulated by the bonded ligand molecules, matches ATP in terms of size, charge, and geometry. Functionalized MCM-41 forms nanosized cavities lined with binding sites on the surface of the solid, into which ATP can effectively fit. Consequently, a larger number of ammonium-ATP interactions can be established in MCM-41 than in silica fume matrices.

These results, together with the detection limit determined for **S2** in the present study, clearly demonstrate that the specific topology of the mesoporpous nanoparticle support significantly enhances ATP detection in terms of both signal intensity and detection limit.

### Photophysical Rationale of the Sensors **S2** and **S3**

Organic π-fluorophores, such as anthracene, have a strong tendency to form H- and J-type aggregates in highly concentrated solutions.^56,57^ The photophysical properties resulting from close [π···π] stacking often differ from those observed when the chromophores exist as isolated units in dilute solutions.^58^ Excimer/exciplex emissions often exhibit bathochromic shifts, which are associated with exciton delocalization across two or more molecules.^59^ Upon addition of ATP, the new emission band at 520 nm in the fluorescence spectra of **S2** and **S3** (Figure 4A,B) can be assigned to ATP-induced excimer formation.^55^ This dual-response behavior is attributed to specific molecular interactions between the sensors and ATP.

At neutral pH, the amine group in **S3** and the hydrazino-2-imidazoline moiety in **S2** are protonated according to their reported p*K*_a_ values.^53,60^ Moreover, based on reported p*K*_a_ values at physiological pH (∼7.0–7.4), the phosphate groups of ATP, ADP, and AMP are largely deprotonated, resulting in approximate net charges of −3 to −4, −2 to −3, and 0 to −1, respectively.^61–63^ The amine groups of **S2** and **S3**, as well as the imidazole-hydrazone moiety of **S2**, can interact with the triphosphate moiety of the ATP through a combination of electrostatic attraction and hydrogen bonding. Furthermore, the central anthracene fluorophore in both sensors may engage in π–π stacking and hydrophobic interactions with the adenine nucleobase, thereby enhancing binding affinity and modulating fluorescence response.^53,55^

Figure S7 presents the molecular electrostatic potential (MEP) analysis for **S2**, **S3**, and ATP molecules.^64^ The MEP is related to electron density and is useful for identifying potential sites for electrophilic and nucleophilic attack, as well as hydrogen bonding interactions.^65^ In this method, regions with more positive potential (blue) tend to interact with nucleophilic species, whereas more negative regions (red) preferentially interact with electrophilic species. In Figure S7, the color scale ranges from −2 × 10^–2^ (red) to 2 × 10^–2^ (blue). Possible electrostatic interactions can be observed between N–H groups (deep blue) on solids **S2** and **S3** and oxygen atoms of the phosphate groups (deep reddish) of ATP.

To further elucidate the interaction mechanism between ATP and solids **S2** and **S3**, density functional theory (DFT) calculations were performed using the Gaussian 16W software package. Geometry optimizations were carried out at the B3LYP/6-31G­(d) level, while interaction energies were evaluated using the ωB97XD/6-31G­(d) functional to account for dispersion effects.^66,67^ Solvent effects were included using the integral equation formalism polarizable continuum model (IEFPCM) with water as the solvent. The calculations were conducted using models incorporating the functionalized groups grafted onto the mesoporous silica nanoparticle framework (Figure S8). Optimized structures of ATP interacting with **S2** and **S3** are shown in Figures S9A,B and S9C,D, respectively. The results reveal strong π–π stacking interactions between the adenine moiety of ATP and the anthracene units of the solids in both the gas phase (**S2**: 3.25–3.46 Å; **S3**: 3.45–3.56 Å) and aqueous medium (**S2**: 3.73–3.58 Å; **S3**: 3.58–3.43 Å). In aqueous media, Figure S9 also indicates the possibility of ionic hydrogen bonding between phosphate groups of ATP and **S2** and **S3**, respectively (Figure S9B: **S2-**ATP: 1.96 Å, Figure S9D: **S3-**ATP:2.57 Å).

### Fluorescence Response toward ATP and Related Anions

To evaluate the anion binding selectivity of the sensing materials, additional anions—ADP, AMP, CTP, pyrophosphate, phosphate, fluoride, chloride, acetate, and benzoate—were tested as potential interferents. The fluorescence responses of **S2** and **S3** upon addition of each analyte (1 mM) were recorded and are shown in Figure 5. Normalized fluorescence emissions (*F*_A_ – *F*_0_)/*F*_0_ are shown at ca. 520 and 420 nm, where *F*_A_ is the fluorescence intensity in the presence of the analyte and *F*_0_ is the baseline intensity in its absence. As shown in Figure 5B, sensor **S2** displays a greater fluorescence enhancement at 520 nm for ATP compared to the other anions, while **S3** also shows a higher enhancement for ATP at this wavelength. At 420 nm, **S2** exhibits fluorescence quenching for ATP, CTP, ADP, AMP, pyrophosphate, dihydrogen phosphate, and benzoate to varying degrees, whereas fluoride and chloride produce a small fluorescence enhancement (Figure 5A).

**Figure 5:**
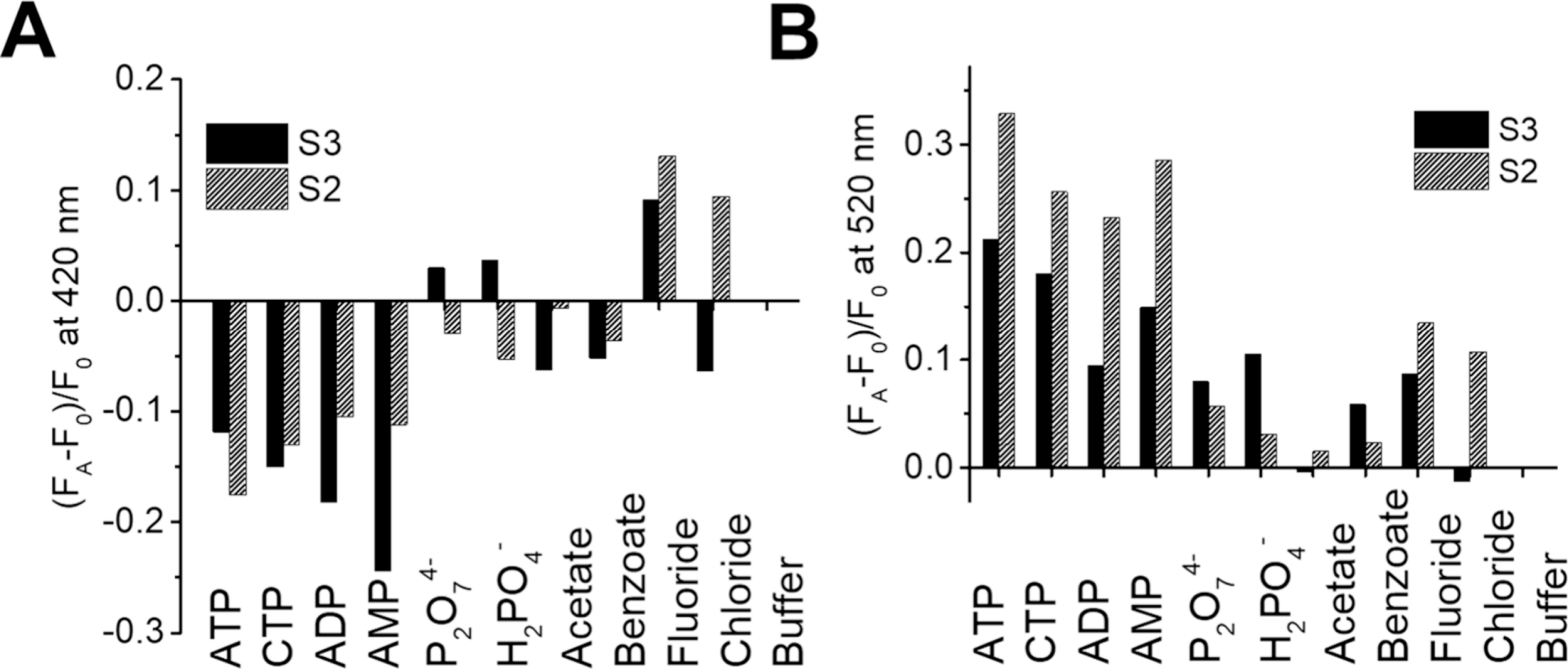
Sensor responses of **S2** (7.48 × 10^–4^ mmol/mg) and **S3** (1.72 × 10^–3^ mmol/mg) upon the addition of ATP, ADP, AMP, CTP, P_2_O_7_^4–^, H_2_PO_4_^–^, AcO^–^, benzoate, F^–^, and Cl^–^ (analyte concentration: 1 mM). (A) Emission at 420 nm and (B) emission at 520 nm (TRIS-HCl pH 7.0, 20 mM).

Although neither **S2** nor **S3** demonstrates strong selectivity for ATP, each generates distinct fluorescence patterns for the different anions tested in the TRIS-HCl buffer (pH 7). These analyte-specific patterns provide the basis for unambiguous identification and classification of the analytes through the development of a cross-reactive sensor array incorporating both MCM-41 nanoparticle-based **S2** and **S3** solids.

### Multivariate Data Analysis Based on LDA and ANN

#### LDA-Based Qualitative Analysis in Buffer and Saliva

The resulting fluorescence patterns were analyzed using LDA, a supervised classification algorithm that employs labeled training data to construct predictive models capable of classifying unknown samples.^68^ LDA maximizes the separation between different multidimensional data classes (e.g., scores for ATP and ADP samples are as different as possible) while minimizing within-class variance (e.g., all ATP samples have similar scores), thus simplifying the interpretation of observed processes (e.g., fluorescence changes). By retaining information about the analyte class during the algorithm’s construction, LDA provides robust discrimination capabilities. LDA score plots enable a visual evaluation of class clustering and the degree of discrimination. LDA was applied using the SYSTAT software package and directly using classical discriminant analyses.

When we applied LDA for eight analytes, the performance of the qualitative analysis in TRIS:HCl buffer solution at pH 7 showed 85% correct classification (Figure S10). To improve the performance, we focused on biologically active phosphate species (ATP, ADP, and AMP) and grouped other anions as nonresponsive (N.R.) as they show a relatively lower sensor response (Figure 5) than ATP, ADP, and AMP. CTP was not tested because unlike adenosine-based nucleotides that govern cellular energy metabolism and could be implicated in PD-related bioenergetic dysfunction, CTP primarily serves biosynthetic roles and is therefore not expected to be mechanistically related to PD. Figure 6A shows the LDA-based discrimination of eight analytes (ATP, ADP, AMP, N.R.: chloride, fluoride, dihydrogen phosphate, acetate, benzoate, and TRIS-HCl buffer) where the first three canonical factors define a multidimensional response space incorporating signals from both **S2** and **S3**. The qualitative assay based on a functionalized MCM-41 sensor array was able to discriminate ATP, ADP, AMP, and other possible interferents in TRIS-HCl with high accuracy (97% correct classification; Figure S11).

**Figure 6:**
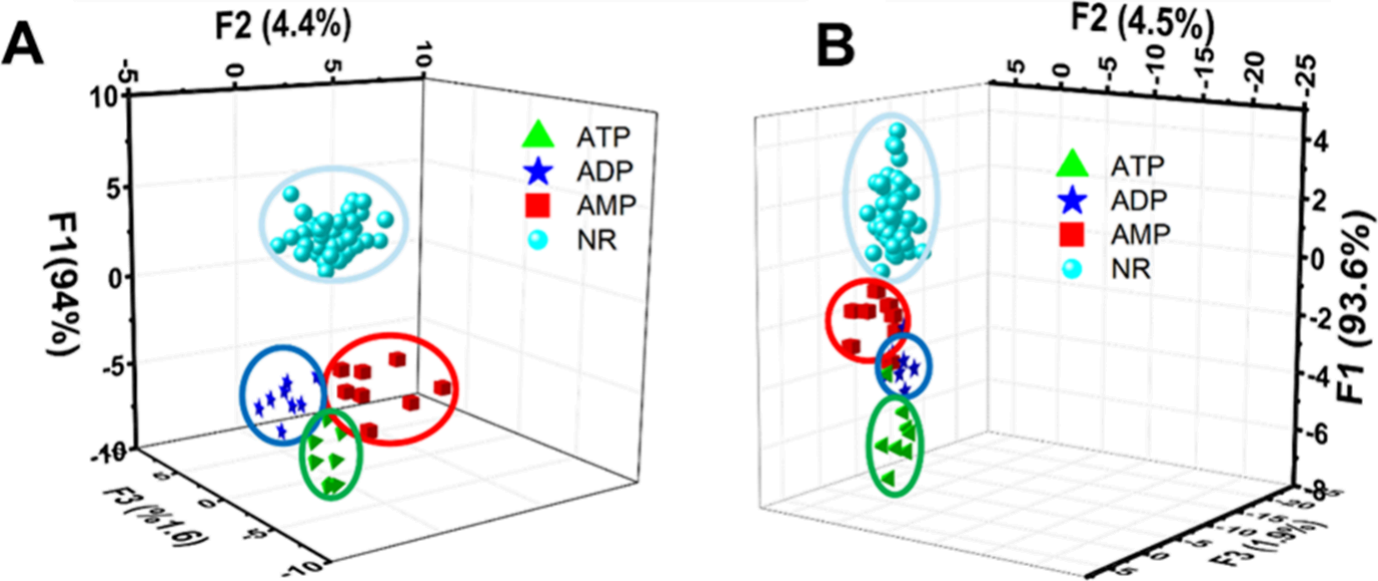
(A) Linear discriminant analysis (LDA) plot of eight analytes in TRIS-HCl buffer (pH 7) (N.R.: chloride, fluoride, benzoate, acetate, dihydrogen phosphate, and buffer) and (B) results of qualitative linear discriminant analysis (LDA) in saliva.

To evaluate the applicability of this sensing strategy in more complex matrices, qualitative (LDA) analysis was extended to saliva samples. Qualitative analysis via LDA achieved 97% correct classification in saliva, with the training data transformed into three canonical scores (Figure 6B and Figure S12).

#### ANN-Based Quantitative Analysis in Buffer and Saliva

While LDA proved to be a simple, efficient, and computationally inexpensive tool for qualitative discrimination, it inherently assumes that the data set follows a Gaussian distribution and is linearly separable.^69^ In practice, chemical sensing data sets often display nonlinear relationships, and the covariance matrices of different classes may not be identical. Under such conditions, more advanced supervised algorithms—such as ANN—offer a distinct advantage for quantitative analysis. Owing to their multilayer architectures and a variety of activation functions, ANNs can model complex, nonlinear dependencies between inputs and outputs, making them particularly well-suited for sensing systems where fluorescence signals may not vary linearly with analyte concentration.^70^ Thus, following the successful qualitative discrimination of analytes by LDA, quantitative determination of ATP in TRIS-HCl buffer (pH 7, 20 mM) was performed using ANN regression analysis.

ANN analysis was performed using the Solo (Eigenvector) software package. Software-controlled ANN parameters available in Eigenvector Solo are given in Table S4. ATP concentrations ranging from 0 to 5 mM were used as the training data set, while two concentrations (0.75 and 1.5 mM) were reserved for validation. Cross-validation confirmed the robustness of the ANN model, yielding acceptable error values (7.8%) and excellent predictive performance, with a coefficient of determination (*R* ^2^ = 1.000), indicating near-perfect linearity between predicted and actual ATP concentrations in TRIS-HCl buffer, at pH 7 (Figure S13). Quantitative ANN analysis in saliva also produced acceptable error values (5.58%) and high predictive accuracy (*R* ^2^ = 0.998) (Figure S14).

### Pattern Recognition-Based Discrimination of Parkinson’s Disease in Saliva

The sensor array was applied to saliva samples to discriminate Parkinson’s disease based on pattern recognition, given that no objective and easily obtainable diagnostic marker is currently available. The resulting fluorescence data were analyzed using LDA to discriminate between the two groups (validation data in Figure S15). A training data set was formed by choosing 5 healthy and 5 PD-positive saliva samples randomly. Training data sets were randomly selected using the sampling tool available in Microsoft Excel’s Data Analysis ToolPak. The rest of the samples were used for prediction, and all samples were tested in quadruplicate. The classification results are summarized in the confusion matrix (Table 2). Using the LDA-based sensor array, 14 out of 19 Parkinson’s disease samples and 15 out of 18 healthy samples were correctly classified.

**Table 2:** Confusion Matrix of Array Sensing of Saliva

	unknown		
predict	healthy	Parkinson’s disease	total
healthy controls	15	3	18
Parkinson’s disease	5	14	19

We also performed ROC curve analysis and reported the results in the Supporting Information (Figure S16). Based on the ROC-derived optimal cutoff, sensitivity and specificity values were calculated, and 95% confidence intervals were estimated assuming a binomial distribution using the Wilson score method. ROC analysis for Parkinson’s disease versus healthy controls yielded an AUROC of 0.76 (95% CI: 0.68–0.84). At the optimal cutoff, the sensitivity was 73.7% (95% CI: 51–88%) and the specificity was 83.3% (95% CI: 61–94%). Confidence intervals were calculated by using the Wilson binomial method.

Although the results obtained from saliva samples are promising, this study should be considered as a proof of concept. Larger and independent cohorts together with additional sensing elements with diverse interaction mechanisms will be required to assess the robustness and clinical applicability of the proposed sensor array.

## Conclusions

In conclusion, we developed an optical sensor array based on two anthracene-modified MCM-41 mesoporous nanoparticle hybrid materials for ATP detection. The mesoporous silica nanoparticles (MCM-41) were functionalized with two different anthracene derivatives: imidazolium-anthracene (**S2**) and aminoanthracene (**S3**). The sensing mechanisms of **S2** and **S3** are governed by a combination of electrostatic attraction and hydrogen bonding. In addition, based on the DFT calculations, π–π interactions between the central anthracene unit and adenine base might also help the recognition of ATP. The functionalization of MCM-41 solids with artificial probes creates more binding sites in nanosized cavities that well fit with the ATP shape, thereby increasing the overall sensitivity. **S2** and **S3** as a sensor array successfully discriminated phosphate-containing species—ATP, ADP, and AMP—with high qualitative accuracy, achieving a 97% correct classification rate in both TRIS-HCl buffer and saliva. Quantitative ATP analysis using artificial neural networks (ANN) demonstrated excellent predictive performance, with strong linear correlations between predicted and actual concentrations in buffer (*R* ^2^ = 1.000) and saliva (*R* ^2^ = 0.998). Furthermore, the developed sensor array was applied to saliva samples collected from individuals with Parkinson’s disease (PD). The array accurately identified 15 of 18 healthy samples (73.7% sensitivity) and 14 of 19 PD samples (83.3% specificity). Although the results obtained from saliva samples are promising, the present study should be considered as a proof of concept. Larger and independent cohorts together with additional sensing elements will be required to assess the robustness and clinical applicability of the proposed sensor array.

## Supplementary Material



## Notes

EDX results, TGA results, LDA results, ^1^H NMR result, ANN result, ROC curve, and clinical characteristics of PD patients (PDF)
