# Supplementary material for: Anthracene-Modified Nanoporous Silica Nanoparticles for ATP Detection and Salivary Diagnostics in Parkinson’s Disease
Source: ACS Appl Nano Mater. 2026 Mar 20;9(13):5683–94. doi: 10.1021/acsanm.5c05223 (PMC13054783; doi:10.1021/acsanm.5c05223)
Supplement: Supplementary file 1 [file an5c05223_si_001.pdf]

SUPPORTING INFORMATION

Anthracene-Modified Nanoporous Silica Nanoparticles for ATP Detection and Salivary Diagnostics in Parkinson's Disease

Elcin Ezgi Ahi[A,B], *Estela Climent*[B,C,D], *Cansu Beyret*[E], *Mehmet Gokhan Caglayan*[E], *Burak Coban*[F], *Rezzak Yilmaz*[F,], *Vicente Martí-Centelles*[B,C,H], *Ramón Martínez-Máñez*[B,C,D,G,H]*, *Mahmut Durmuş*[A]*

A. Department of Chemistry, Gebze Technical University, Gebze, 41400, Kocaeli, Türkiye

B. Instituto Interuniversitario de Investigación de Reconocimiento Molecular y Desarrollo Tecnológico (IDM), Universitat Politècnica de València, Universitat de València, Camino de Vera s/n, 46022, Valencia, Spain

C. CIBER de Bioingeniería Biomateriales y Nanomedicina (CIBER-BBN), Instituto de Salud Carlos III, 46022, Valencia , Spain.

D. Unidad Mixta de Investigación en Nanomedicina y Sensores, Universitat Politècnica de València, Instituto de Investigación Sanitaria La Fe (IIS La Fe), Avenida Fernando Abril Martorell, 106, 46026, Valencia, Spain.

E. Ankara University, Faculty of Pharmacy, Department of Analytical Chemistry, Yenimahalle, 06560, Ankara, Türkiye

F. Department of Neurology, Ankara University, School of Medicine, Ankara, Türkiye. Brain Research Center, Ankara University, Altındağ, 06230, Ankara, Turkey

G. Unidad Mixta UPV-CIPF de Investigación en Mecanismos de Enfermedades y Nanomedicina, Valencia, Universitat Politècnica de València, Centro de Investigación Príncipe Felipe, Avenida Eduardo Primo Yúfera, 3, 46012, Valencia, Spain.

H. Departamento de Química, Universitat Politècnica de València, Camino de Vera s/n, 46022, Valencia, Spain.

E-mail: rmaez@qim.upv.es, durmus@gtu.edu.tr

CONTENT	PAGE
^{1}H NMR spectra of 2-hydrazino-2-imidazoline hydrobromide	S-2
Construction of a microplate for qualitative analysis	S-2
Clinical characteristics of patients with Parkinson's disease	S-3
Thermogravimetric analysis	S-3
The loss of mass calculated from the curve of thermogravimetric analysis	S-4
Elemental composition of **S0**, **S2** and **S3** nanoparticles	S-4
SEM-EDX analysis of **S0-S2-S3**	S-4,S-5
Molecular electronic potential (MEP) of **S2**, ATP, and **S3**	S-6
Optimized structures of **S2** and **S3**	S-7
Linear discriminant analysis (LDA) of eight analytes in TRIS:HCl buffer	S-8
LDA of eight analytes in saliva	S-9
ANN parameters available in Eigenvector SOLO	S-9
ANN calibration curves	S-10
LDA of saliva samples of healthy and Parkinson's persons	S-11
ROC curve analysis for Parkinon's Disease versus healthy controls	S-12

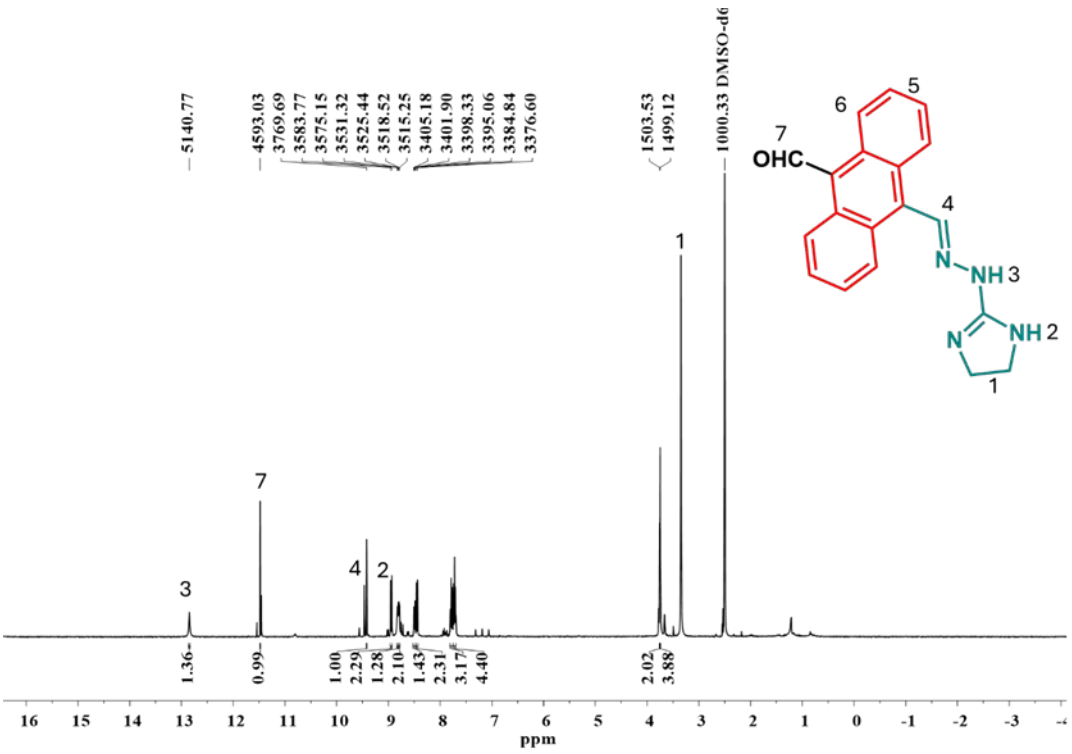

Figure S1: ^{1}H NMR spectra of 2-hydrazino-2-imidazoline hydrobromide (compound **1**)

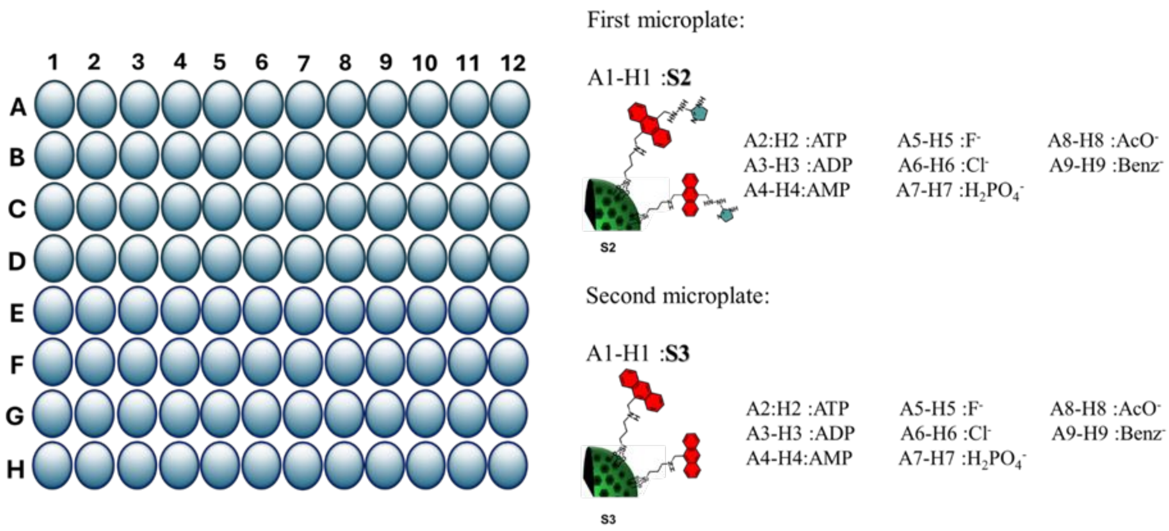

Figure S2: Construction of a microplate for qualitative analysis with sensor arrays of S2 and S3 in TRIS-HCl (pH 7)

Table S1. Clinical characteristics of patients with Parkinson's disease (n=24)

Age, years, mean (SD)	65.0 (12.2)
Male sex, n (%)	8.0 (33.3)
Disease duration, years, median (IQR)	3.0 (5.0)
LEDD, mg/day, mean (SD)	512.8 (542.5)
MDS-UPDRS-I, mean (SD)	11.7 (8.8)
MDS-UPDRS-III, mean (SD)	42.1 (15.8)
Hoehn & Yahr, median (IQR)	2.0 (1.0)

SD, standard deviation; IQR, interquartile range; LEDD, levodopa-equivalent daily dose; MDS-UPDRS, Movement Disorder Society-Unified Parkinson's Disease Rating Scale.

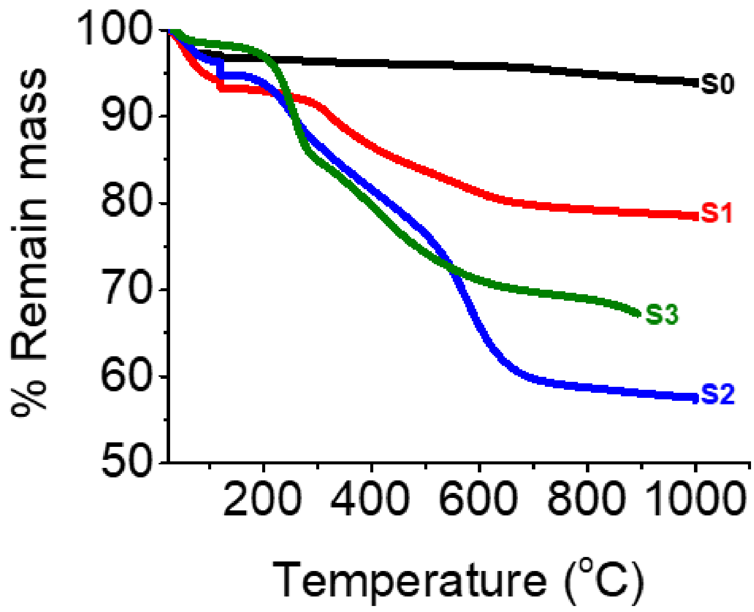

Figure S3: Thermogravimetric analysis for **S0**, **S1, S2** and **S3** solids.

Table S2: The loss of mass calculated from the curve of thermogravimetric analysis for **S0**, **S1**, **S2** and **S3**.

Temperature	**S0** (calcined) (Weight loss%)	**S1** (Weight loss%)	**S2** (Weight loss%)	**S3** (Weight loss%)
25 - 165 °C	3.29	6.82	5.38	1.75
165 - 700 °C	1.35	13.41	35	29.42
700 - 900 °C	1.35	1.36	1.58	1.56

Table S3: Elemental composition of **S0**, **S2** and **S3** nanoparticles determined by elemental analysis (Wt%) (See **Figure 3A-C**)

	**% N** (Wt%)	**%C** (Wt%)	**%O** (Wt%)	**% Si** (Wt%)
**S0**	-	-	59.48±0.33	40.52±0.20
**S2**	4.82±0.07	29.35±0.01	38.92±0.01	24.25
**S3**	4.63±0.08	40.81±0.01	37.14±0.01	16.12±0.01

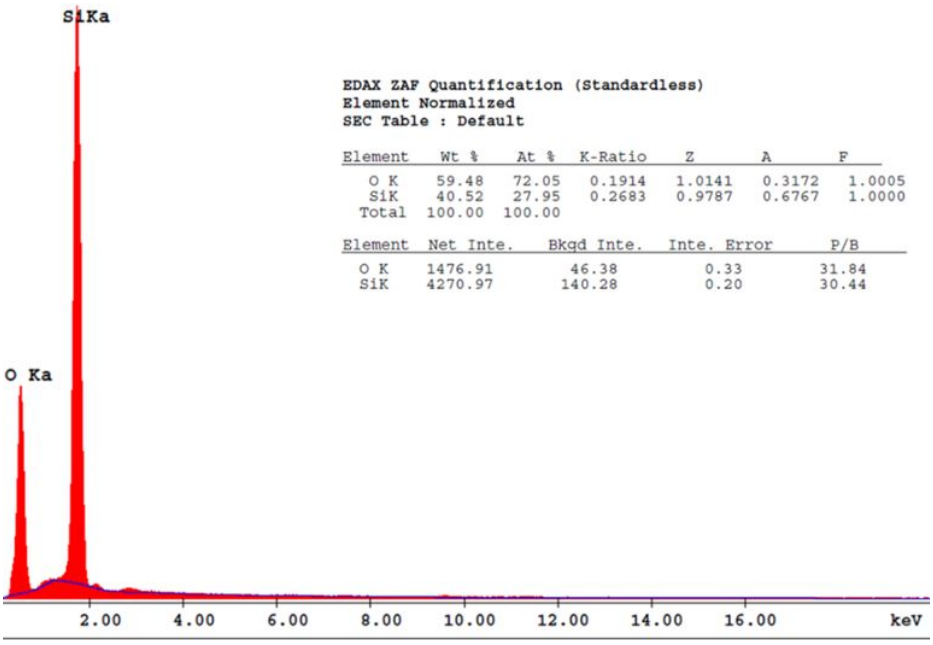

Figure S4: SEM-EDX analysis of **S0**

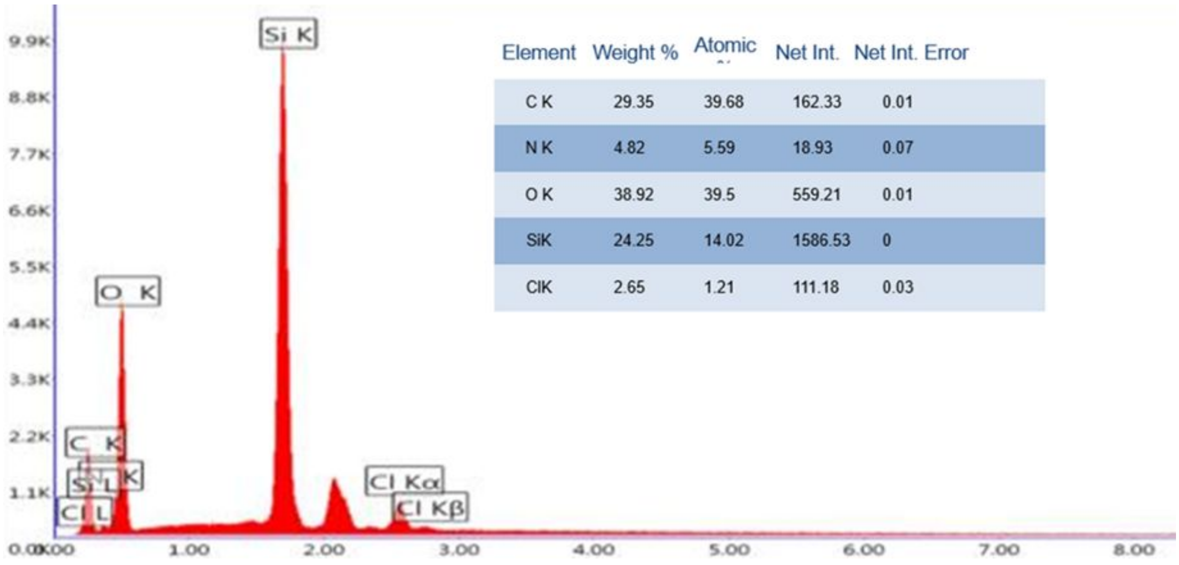

Figure S5: SEM-EDX analysis of **S2**

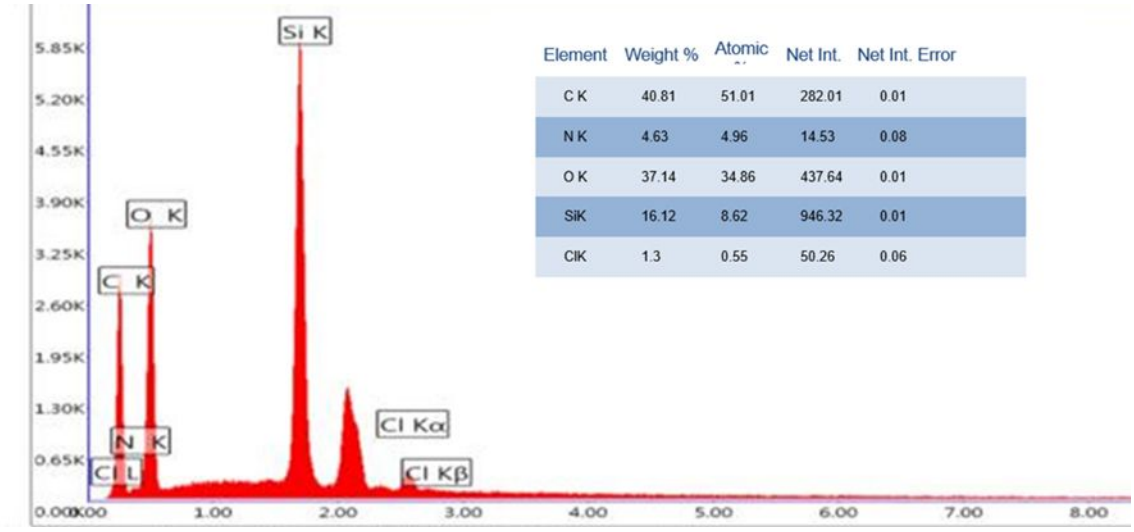

Element	Weight %	Atomic	Net Int.	Net Int. Error
C K	40.81	51.01	282.01	0.01
N K	4.63	4.96	14.53	0.08
O K	37.14	34.86	437.64	0.01
SiK	16.12	8.62	946.32	0.01
ClK	1.3	0.55	50.26	0.06

Figure S6: SEM-EDX analysis of **S3**

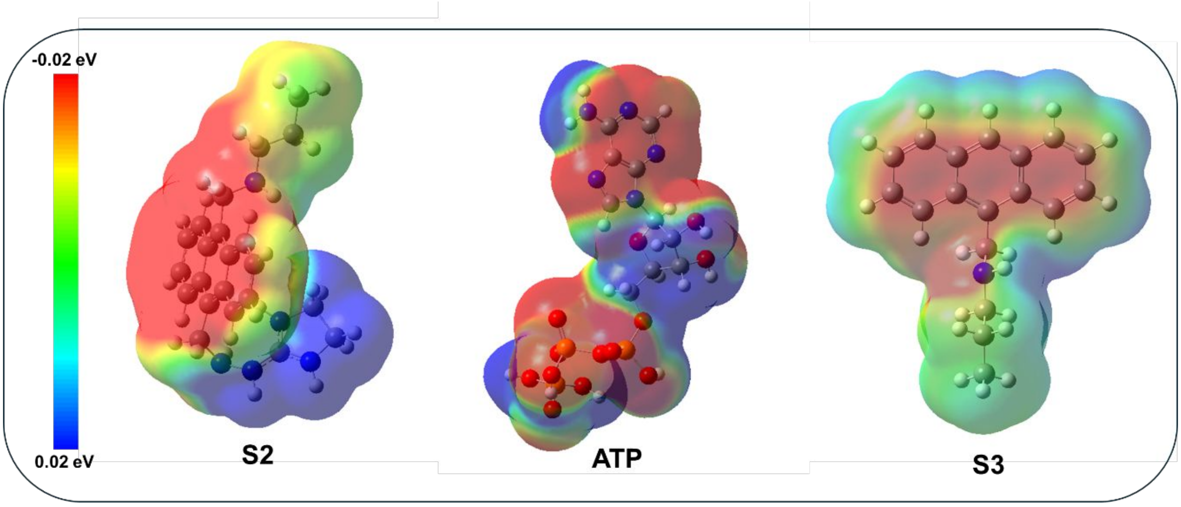

Figure S7: Molecular electronic potential (MEP) of **S2**, ATP, and **S3** to show negative and positive sites of molecules in aqueous media, respectively. The color scale on the left shows from red to blue which indicates negative and positive sites, respectively.

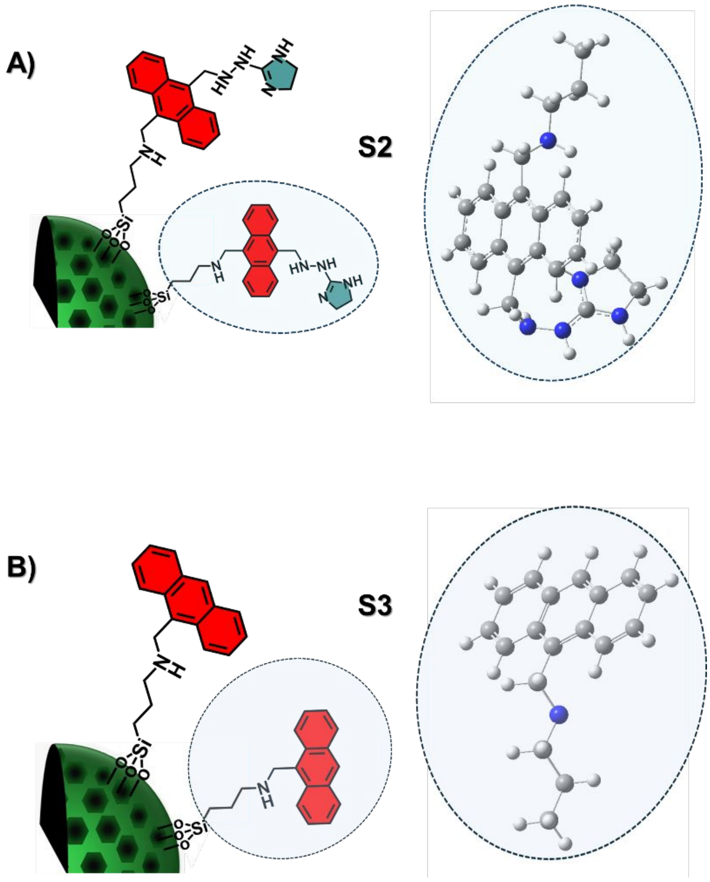

Figure S8: The respective structure of the functional molecule that was considired in density functional theory (DFT) calculations studies

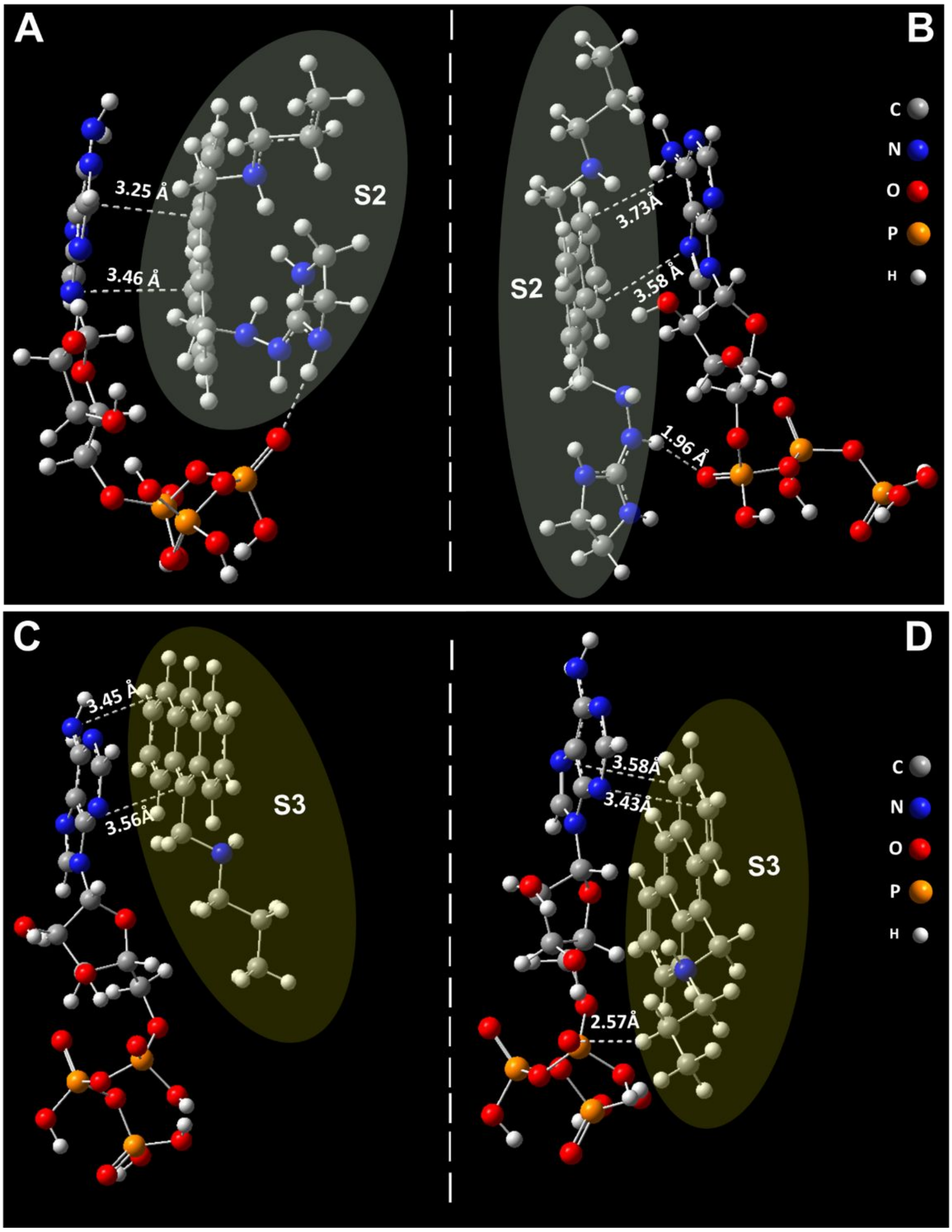

Figure S9. Optimized structures of **A**: S2 with ATP in gas phase; **B**: S2 with ATP in water; **C**: S3 with ATP in gas phase; and **D**: S3 With ATP in water phase.

Classification Matrix (Cases in row categories classified into columns)

	ADP	AMP	ATP	Acetate	Benzoate	Blank	Chloride	Fluoride	Phosphate	%correct
ADP	8	0	0	0	0	0	0	0	0	100
AMP	0	8	0	0	0	0	0	0	0	100
ATP	0	0	8	0	0	0	0	0	0	100
Acetate	0	0	0	7	0	0	1	0	0	88
Benzoate	0	0	0	0	6	0	0	0	2	75
Blank	0	0	0	0	0	8	0	0	0	100
Chloride	0	0	0	0	1	1	5	1	0	63
Fluoride	0	0	0	2	0	0	0	6	0	75
Phosphate	0	0	0	1	1	1	0	0	5	63
Total	8	8	8	10	8	10	6	7	7	85

Jackknifed Classification Matrix

	ADP	AMP	ATP	Acetate	Benzoate	Blank	Chloride	Fluoride	Phosphate	%correct
ADP	8	0	0	0	0	0	0	0	0	100
AMP	1	5	2	0	0	0	0	0	0	63
ATP	1	0	7	0	0	0	0	0	0	88
Acetate	0	0	0	3	0	0	4	1	0	38
Benzoate	0	0	0	0	4	0	1	0	3	50
Blank	0	0	0	0	0	5	3	0	0	63
Chloride	0	0	0	2	2	2	1	1	0	13
Fluoride	0	0	0	1	0	0	1	6	0	75
Phosphate	0	0	0	2	4	1	0	0	1	13
Total	10	5	9	8	10	8	10	8	4	56

Eigenvalues

34,704	1,725	0,845	0,528	0,124	0,060	0,020	0,005

Figure S10: Linear discriminant analysis (LDA) of eight analytes in TRIS:HCl buffer (pH 7)

Classification Matrix (Cases in row categories classified into columns)

	ADP	AMP	ATP	N.R.	%correct
ADP	8	0	0	0	100
AMP	0	7	1	0	88
ATP	1	0	7	0	88
N.R.	0	0	0	48	100
Total	9	7	8	48	97

Jackknifed Classification Matrix

	ADP	AMP	ATP	N.R.	%correct
ADP	6	1	1	0	75
AMP	1	6	1	0	75
ATP	1	0	7	0	88
N.R.	0	0	0	48	100
Total	8	7	9	48	93

Eigenvalues

23,661	1,117	0,391

Canonical Correlations

0,980	0,726	0,530

Cumulative Proportion of Total Dispersion

0,940	0,984	1,000

Figure S11: Linear discriminant analysis (LDA) of eight analytes in TRIS:HCl buffer (pH 7) (N.R. : chloride, fluoride, benzoate, acetate, dihydrogen phosphate, buffer)

Classification Matrix (Cases in row categories classified into columns)

	ADP	AMP	ATP	NR	%correct
ADP	8	0	0	0	100
AMP	1	7	0	0	88
ATP	0	0	8	0	100
NR	1	0	0	47	98
Total	10	7	8	47	97

Jackknifed Classification Matrix

	ADP	AMP	ATP	NR	%correct
ADP	7	1	0	0	88
AMP	3	4	0	1	50
ATP	2	0	5	1	63
NR	2	3	0	43	90
Total	14	8	5	45	82

Eigenvalues

8,085	0,387	0,165

Canonical Correlations

0,943	0,528	0,376

Cumulative Proportion of Total Dispersion

0,936	0,981	1,000

Figure S12: Linear discriminant analysis (LDA) of eight analytes in saliva (pH 7) (N.R.: chloride, fluoride, benzoate, acetate, dihydrogen phosphate, buffer)

Table S4. ANN parameters available in Eigenvector SOLO

Preprocessing:	Autoscaling
Cross-validation method:	Venetian blinds
Left-out data per split:	minimum 8%, maximum 13%, average 10%
Number of data splits:	12
Samples per blind:	1
Maximum number of nodes:	1

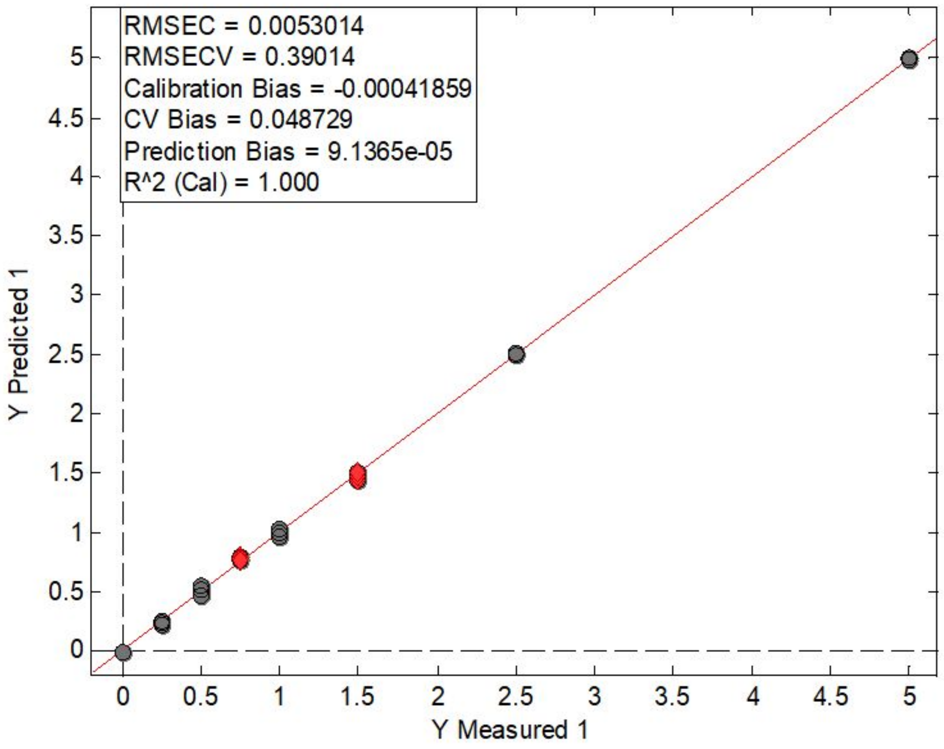

Figure S13: Artificial neural networks (ANN) calibration curve (dynamic range: 0.25-5 mM) in TRIS-HCl buffer (pH 7, 20 mM)

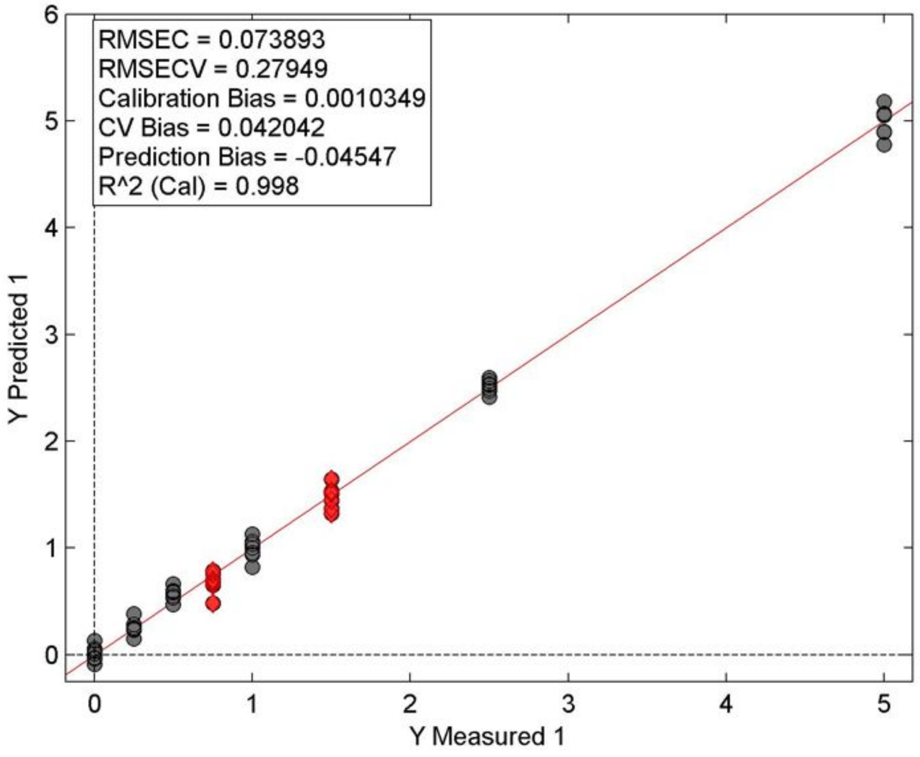

Figure S14: Artificial neural networks (ANN) calibration curve (dynamic range: 0.25-5 mM) in saliva

Classification Matrix (Cases in row categories classified into columns)

	HEALTHY	PARKINSON	%correct
HEALTHY	16	4	80
PARKINSON	5	15	75
Total	21	19	78

Classification of Cases with zero weight or frequency

	HEALTHY	PARKINSON	%correct
HEALTHY	0	0	0
PARKINSON	0	0	0
%correct	0	0	0

Jackknifed Classification Matrix

	HEALTHY	PARKINSON	%correct
HEALTHY	15	5	75
PARKINSON	7	13	65
Total	22	18	70

Eigenvalues

0.652

Canonical Correlations

0.628

Cumulative Proportion of Total Dispersion

1.000

Figure S15: Linear Discriminant Analysis (LDA) of saliva samples of healthy and Parkinson's persons

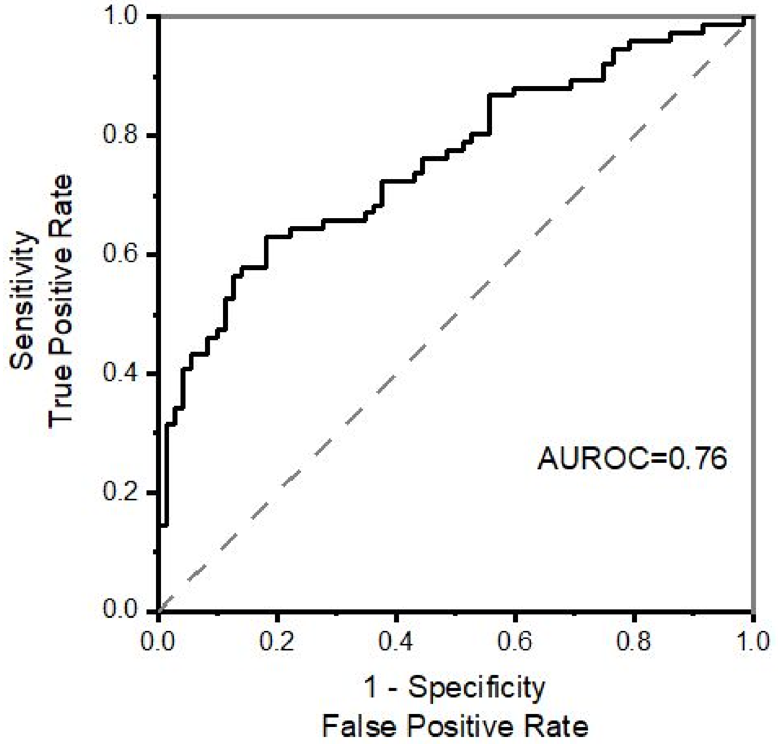

Figure S16: ROC curve analysis for Parkinon's Disease versus healthy controls
